# Supplementary material for: Patterns of Intron Gain and Loss in Fungi
Source: PLoS Biol. 2004 Nov 30;2(12):e422. doi: 10.1371/journal.pbio.0020422 (PMC532390; doi:10.1371/journal.pbio.0020422)
Supplement: Table S1 — Also available at http://genes.mit.edu/NielsenEtAl/. (4.3 MB ZIP). [file pbio.0020422.st001.zip › NielsenEtAl/html/1237.html]

AN2525.1.NCU03607.1.MG09501.1.FG10119.1


```
 CLUSTAL W (1.82) Multiple Sequence Alignments - Introns Inserted


Sequence 1: MG09501.1	403 aa
Sequence 2: FG10119.1	398 aa
Sequence 3: NCU03607.1	388 aa
Sequence 4: AN2525.1	361 aa
Alignment Length: 415 aa
Number Identitical Residues: 155 aa
Alignment Score (without introns) 8248


MG09501.1 	MADPVTSPPLERASIFAAHELIKPYVHRTPVLTNATLTALASTPRTAEDLRGTRWADRAD
NCU03607.1	MADPSTTPPLTRASVLAAHSLIKPYIHETPVLTNTTFDQLASTPRTPEELQGTEWEATER
FG10119.1 	MADISKTPPLTRDSVIEAHKLIGQYVHYTPVLTNRTITELASTPRTAEELEGTRYAGRT-
AN2525.1  	MAPQLPTP----NSIQEAYTLITPYIHRTPLLTNRTLNAIASARQSPDALRGTPFEGST-
          	**    :*     *:  *: **  *:* **:*** *:  :**: ::.: *.** :     

MG09501.1 	RPARPVLRLWFKCENFQRIGAFKPRGAFHAVERLKLIPGWLEGQGRTNGVVTASSG1NHA
NCU03607.1	-PANPKIRFWFKCENFQRIGAFKARGAFHAVERLKQTEG-LEG-LKKGGVVTHSSG1NHA
FG10119.1 	-PARPVLRLWFKCENMQRIGAFKARGAFHAVERLKQEPGWLEGGGKEKGVVTHSSG1NHA
AN2525.1  	-PAHPKINFFFKCENFQRIGAFKARGAFHALLRLIEREG-----------------~-EE
          	 **.* :.::*****:*******.******: **    *                   . 

MG09501.1 	Q~ALALAARTAGIPAHIVIPSIAPAPKLAATKALGATIYRSGSTGAEREAVAAKVVADTG
NCU03607.1	Q~ALSLAARENGIPAHIVMPTISPPPKIAATKGYGANITFSGSTSTEREAVTREVIEKTG
FG10119.1 	Q~ALALAARESGINAHIVMPEISNPKKIAGTKGYGARVIFSGSTSTEREAMAEKVIAETG
AN2525.1  	N0ALALASSTLGIPAYIVMPKISTPSKIAGTQSHGAEVIFSGSTSVEREAVVADVQARTG
          	: **:**:   ** *:**:* *: . *:*.*:. ** :  ****..****:. .*   **

MG09501.1 	GRYIPPYDH~PDIILGQGTLGLEMQEQVEALMAGGDAAATGGDFTRRGQTSGTQGQAGGQ
NCU03607.1	ARLVPPYDH1PDIILGQGTAALELQRQVAASLST------------SGTTTTTNRRR---
FG10119.1 	ARLIPPYDH~PDIMLGQGTMGLEFQQQVAELMTT-DAVVDGQRKLFRAPSTGRDNEAPRT
AN2525.1  	AILVPPYDD~YHIICGQGTTGLEMHAQYLDAVRE------------KPELSVHESESPSA
          	.  :****.  .*: **** .**:: *    :                  :  : .    

MG09501.1 	KK---GLNAIIAPCGGGGMLSGVALSCEGTG---------IRVFGAEPEFQGADECKRSF
NCU03607.1	------LNAIITPCGGGGLLSGTALACSDLSPSDPSTPGPILVFGAEPSFSGADDGRRGY
FG10119.1 	KKEKPGLNAIMTPLGGGGMLSGVALSCEGTG---------IRVFGSEPSFEGADDGKRGY
AN2525.1  	REG---LDAVITPIGGGGLNSGVATFFSDKT---------TKVFGAEPSFEGADDCRRGL
          	 .    *:*:::* ****: **.*   ..             ***:**.*.***: :*. 

MG09501.1 	EAGKRIDTVKSLTIADGLRTAVGKYPWSIIYERRLVSQMFNVTEDEIKDALRLILERFKL
NCU03607.1	YSGTRIESVKSLTIADGLRTPLGAYPWSIIYERKLVAGMYSVGEEEIKKALRLVYERMKV
FG10119.1 	ESGSRITAVKTLTVADGLRTPVGAHPWSVIYERRLVSGIYSVTEDEILSAMKLVFERFKL
AN2525.1  	DAGQRVESVKTLTIADGLRTPVGVLNWEVISDKSKVAGVFSVTEEQIKAAMRLVLERMKV
          	 :* *: :**:**:******.:*   *.:* ::  *: ::.* *::*  *::*: **:*:

MG09501.1 	VVEPSAAVPVAVALFNEDFRAMVETEAGENGWDLGIVLSGGNVSLEMLGKLFPEGLPA
NCU03607.1	VVEPSAVVGLAVALFNEEFRSMVEREGGEEGWDLGVVFSGGNVELAALGRLFGEE---
FG10119.1 	VVEPSACVPLAVALYDEDFRSMVEKEAGEEGWDLGLVFSGGNVGLDAIGKLFA-----
AN2525.1  	VVEPSAVVGLAVCLYNEEFRRLVEREAP-AGWDVGIVFSGGNTTVEAIGKLFG-----
          	****** * :**.*::*:** :** *.   ***:*:*:****. :  :*:**
```
